# Identification of Cholecystokinin by Genome-Wide Profiling as Potential Mediator of Serotonin-Dependent Behavioral Effects of Maternal Separation in the Amygdala

**DOI:** 10.3389/fnins.2019.00460

**Published:** 2019-05-10

**Authors:** Magdalena T. Weidner, Roy Lardenoije, Lars Eijssen, Floriana Mogavero, Lilian P. M. T. De Groodt, Sandy Popp, Rupert Palme, Konrad U. Förstner, Tatyana Strekalova, Harry W. M. Steinbusch, Angelika G. Schmitt-Böhrer, Jeffrey C. Glennon, Jonas Waider, Daniel L. A. van den Hove, Klaus-Peter Lesch

**Affiliations:** ^1^Department of Psychiatry and Neuropsychology, School for Mental Health and Neuroscience (MHeNs), Maastricht University, Maastricht, Netherlands; ^2^Division of Molecular Psychiatry, Laboratory of Translational Neuroscience, Center of Mental Health, Department of Psychiatry, University of Würzburg, Würzburg, Germany; ^3^Department of Psychiatry and Psychotherapy, Medical Center – University of Freiburg, Faculty of Medicine, University of Freiburg, Freiburg, Germany; ^4^Department of Psychiatry and Psychotherapy, Universitätsmedizin Göttingen, Georg-August-Universität, Göttingen, Germany; ^5^Department of Psychiatry, McLean Hospital, Harvard Medical School, Belmont, MA, United States; ^6^Departments of Bioinformatics, Psychiatry & Neuro Psychology, NUTRIM School of Nutrition and Translational Research in Metabolism, Maastricht University, Maastricht, Netherlands; ^7^Donders Institute for Brain, Cognition and Behaviour, Radboud University, Nijmegen, Netherlands; ^8^Department of Biomedical Sciences, University of Veterinary Medicine, Vienna, Austria; ^9^Core Unit Systems Medicine, Institute for Molecular Infection Biology, University of Würzburg, Würzburg, Germany; ^10^ZB MED – Information Centre for Life Sciences, Cologne, Germany; ^11^TH Köln, Faculty of Information Science and Communication Studies, Cologne, Germany; ^12^Laboratory of Psychiatric Neurobiology, Institute of Molecular Medicine, I. M. Sechenov First Moscow State Medical University and Institute of General Pathology and Pathophysiology, Moscow, Russia; ^13^Center of Mental Health, Department of Psychiatry, Psychosomatics and Psychotherapy, University Hospital of Würzburg, Würzburg, Germany

**Keywords:** serotonin, maternal separation, mouse, emotional behavior, DNA methylation, RNA expression

## Abstract

Converging evidence suggests a role of serotonin (5-hydroxytryptamine, 5-HT) and tryptophan hydroxylase 2 (TPH2), the rate-limiting enzyme of 5-HT synthesis in the brain, in modulating long-term, neurobiological effects of early-life adversity. Here, we aimed at further elucidating the molecular mechanisms underlying this interaction, and its consequences for socio-emotional behaviors, with a focus on anxiety and social interaction. In this study, adult, male *Tph2* null mutant (*Tph2*^-/-^) and heterozygous (*Tph2*^+/-^) mice, and their wildtype littermates (*Tph2*^+/+^) were exposed to neonatal, maternal separation (MS) and screened for behavioral changes, followed by genome-wide RNA expression and DNA methylation profiling. In *Tph2*^-/-^ mice, brain 5-HT deficiency profoundly affected socio-emotional behaviors, i.e., decreased avoidance of the aversive open arms in the elevated plus-maze (EPM) as well as decreased prosocial and increased rule breaking behavior in the resident-intruder test when compared to their wildtype littermates. *Tph2*^+/-^ mice showed an ambiguous profile with context-dependent, behavioral responses. In the EPM they showed similar avoidance of the open arm but decreased prosocial and increased rule breaking behavior in the resident-intruder test when compared to their wildtype littermates. Notably, MS effects on behavior were subtle and depended on the *Tph2* genotype, in particular increasing the observed avoidance of EPM open arms in wildtype and *Tph2*^+/-^ mice when compared to their *Tph2*^-/-^ littermates. On the genomic level, the interaction of *Tph2* genotype with MS differentially affected the expression of numerous genes, of which a subset showed an overlap with DNA methylation profiles at corresponding loci. Remarkably, changes in methylation nearby and expression of the gene encoding cholecystokinin, which were inversely correlated to each other, were associated with variations in anxiety-related phenotypes. In conclusion, next to various behavioral alterations, we identified gene expression and DNA methylation profiles to be associated with TPH2 inactivation and its interaction with MS, suggesting a gene-by-environment interaction-dependent, modulatory function of brain 5-HT availability.

## Introduction

The serotonin (5-hydroxytryptamine; 5-HT) system is one of the brain’s key neuromodulatory systems and as such involved in the regulation of manifold behaviors (Lesch et al., 2012; Paul and Lowry, 2013; Ottenhof et al., 2018). Mice, carrying a constitutive, genetic tryptophan hydroxylase 2 (TPH2) inactivation (Gutknecht et al., 2008), which results in a brain 5-HT reduction of approximately 90% (Gutknecht et al., 2012), were found to display a distinctive behavioral phenotype (Mosienko et al., 2015). Lifelong 5-HT deficiency is associated with altered anxiety-related behaviors, locomotor activity and social behavior. Next to its direct effects on behavior, 5-HT system function is known to modulate the effects of aversive experiences throughout life (Bennett et al., 2002; Caspi et al., 2002, 2003; Champoux et al., 2002; van den Hove et al., 2011; Gutknecht et al., 2015; Sachs et al., 2015; Wong et al., 2015).

Stress throughout development and further along the lifespan has the capacity to affect the stress response and stress-related behaviors later in life by reprogramming the reactivity of limbic brain areas (Lupien et al., 2009). Stress-induced adaption in limbic function is related to alterations in the 5-HT system (Márquez et al., 2013). Accordingly, genetic TPH2 inactivation induces distinct reactivity of various brain regions (Waider et al., 2017; Auth et al., 2018). Complete brain 5-HT deficiency is concomitant with an altered reactivity of basal amygdala, paraventricular nucleus (PVN) and periaqueductal gray (PAG) to diverse challenges. Moreover, stress was found to affect factors of the 5-HT system directly. Exposure to maternal separation (MS) is associated with increased monoamine oxidase a (*Mao-a*) expression in striatum and brain stem (Wong et al., 2015), decreased 5-HT immunoreactivity in the hypothalamus (Veenema et al., 2006) as well as increased *Tph2* expression in the dorsal raphe (DR), in the context of social challenges (Gardner et al., 2009). Thus, while the 5-HT system modulates the effects of stress, stress directly affects 5-HT system function, reflecting an interaction of genetic and environmental factors. At the core of this interaction, the 5-HT system acts as a modulatory interface, regulating the effects of early experiences. 5-HT system function, during the early postnatal period, is vital for the formation and fine tuning of 5-HT projection connectivity in several limbic brain regions. Migliarini et al. (2013) showed that lack of 5-HT reduces the density of serotonergic fibers in the suprachiasmatic nucleus, the hypothalamus and the thalamic PVN, while it increases the density of 5-HT fibers in nucleus accumbens and hippocampus. A suggested potential mechanism for this highly specific connectivity pattern is a 5-HT-dependent regulation of brain derived neurotrophic factor (*Bdnf*). In support of this view, *Bdnf* expression was linked to early, 5-HT-induced epigenetic modifications at the *Bdnf* gene, (Boulle et al., 2016). Another study investigating the involvement of 5-HT in modulating the epigenetic landscape, indicated that 5-HT signaling during early life facilitates the recruitment of chromatin-remodeling complexes, and transcription factors, such as cAMP response element binding protein (CREB) binding protein and early growth response protein 1 (Hellstrom et al., 2012), resulting in an altered epigenetic regulation of stress-relevant genes such as the glucocorticoid receptor and, consequently the activity of the stress axis (Zhang et al., 2013). Of note, most effects of early-life adversity are sex-specific with regard to behavior, stress responses and 5-HT system mediation (McCormick et al., 1995; Veenema et al., 2007; García-Cáceres et al., 2010; van den Hove et al., 2014; Hiroi et al., 2016).

Taken together, an interaction of 5-HT system function and early-life adversity has already been shown in several studies. However, an investigation of the molecular correlates of the altered behavior, with a focus on socio-emotional behavior and in the context of epigenetic regulation, has not yet been performed. In the present study, we aimed to further elucidate the role brain 5-HT plays in the epigenetic mediation of early, postnatal adversity and the effects this interaction exerts on the behavioral phenotype, with a focus on territorial, social behaviors, including aggression, in adult male mice. To reveal molecular mechanisms that are vital for the integration of environmental cues and stress response, we targeted the amygdala, as one of the core components controlling limbic-mediated, emotional processes, which is thoroughly innervated and modulated by 5-HT projections (Asan et al., 2013), for the genome-wide analysis of RNA expression and DNA methylation.

## Materials and Methods

### Animals and Procedures

All experiments were performed in accordance with the European Parliament and Council Directive (2010/63/EU) and approved by local authorities (Government of Lower Franconia, Würzburg: 55.2-2531.01- 57/12). All efforts were made to minimize numbers and suffering of animals.

Mice of the parental generation were housed in sex-specific groups of 2–7 under a 14 h/10 h light-dark cycle, with lights on at 7 AM–9 PM, in climate-controlled rooms (21 ± 1°C, humidity 45–55%). Standard rodent chow and water were available *ad libitum*. For breeding purposes, 22 male and 44 female *Tph2*^+/-^ mice, backcrossed on C57BL/6N for more than 15 generations, and approximately 3 months of age, were put together in standard (267 × 207 × 140 mm) polysulfone cages (Tecniplast Deutschland GmbH, Hohenpeissenberg, Germany) with woodchips and standard nesting material in a 1:2 breeding design. The number of breeding pairs was calculated based on the targeted group size of 12 animals per genotype and condition, and based on the Hardy-Weinberg equilibrium, a 1:1 sex-ratio, and an estimated breeding success rate of 75%. All mating pairs were separated after 5 days and females that had a vaginal plug at least once were housed individually from then onward. Females were weighed before mating and 4, 7 and 10 days after separation. Animals that did not show any weight gain over the first 10 days after separation were mated again, with different males. From 14 days after separation of the breeding pairs, nests were checked for pups once per day. The day of birth was declared postnatal day (P)0. No cage changes were performed until P5. Litter size was not normalized at birth for amount of pups of various genotypes, sex or total litter size, owed to the higher mortality of *Tph2* null mutant (*Tph2*^-/-^) offspring (Alenina et al., 2009). Furthermore, due to their growth retardation, the determination of sex was more difficult in *Tph2*^-/-^ animals, in particular before the age of weaning. Thus, to ensure a sufficient amount of *Tph2*^-/-^ males no adjustments were made pre-weaning. Only litters of 5 or more pups were included. Thus, litter size of included litters ranged from 5 to 11 pups per litter, with an average litter size of 8.1 ± 0.3 pups per litter. At P2, litters were randomly assigned to MS and control condition. 23 litters and their respective nesting material were removed from the maternal cage for 3 h/day P2 through 15, while dams remained in their respective cages. Of 23 MS litters 6 were excluded post-weaning, due to imbalanced sex-ratio and/or to avoid litter effects of isolated genotypes, i.e., for example litters with only *Tph2*^+/-^ males, leaving 17 MS litters. An equal number of litters (17) had been subjected to standard facility rearing and served as control. Pups were weaned at P24 ± 2. For all following procedures, we used only male offspring. After weaning, mice were housed individually in standard cages under a 12 h light-dark cycle, with lights on 1 AM–1 PM. Physiological parameters of mothers and pups are reported in the Supplementary Information (Data Sheet 1). We found no MS effect on maternal weight, relative pup weight or pup survival and observed a lower weight in *Tph2*^-/-^ animals throughout adulthood (Supplementary Tables 1, 2; Data Sheet 1; Weidner, 2018).

Adult males of both control and MS litters were subjected to behavioral screening starting at approximately 2 months of age. All tests were conducted during the dark phase. In brief, mice were tested for anxiety-related behavior, using the dark-light box (DLB) test (Crawley and Goodwin, 1980; Onaivi and Martin, 1989) followed by open-field (OF) test under red-light conditions to determine locomotor activity (Post et al., 2011) and elevated plus-maze (EPM) test as another anxiety test (Lister, 1987; Post et al., 2011; Gutknecht et al., 2015). Mice were tracked using infrared light from below the respective apparatus (Post et al., 2011). Trials were recorded from above, using an infrared-sensitive CCD camera. Behavioral analysis was performed, using VideoMot2 tracking software (TSE Systems, Bad Homburg, Germany). Each animal was allowed to rest for at least 6 days between trials. The experimental procedures and measures of DLB, EPM and OF are described in more detail in the Supplementary Methods (Data Sheet 1). Following the anxiety-related behavioral test battery animals were tested in a repeated resident-intruder test (RIT). The RIT is an aggression test, which is based on the naturally occurring territoriality, observed in male mice. The used protocol was adapted from a protocol employed in rats (Koolhaas et al., 2013), as described in the following. In preparation of the test, in order to keep olfactory cues stable and, thus, reinforce natural territoriality in the residents, a small amount of soiled sawdust was carried over at every cage change. From 5 days prior to testing until 1 day after testing, cages were not changed. Intruder males were approximately 2 months old DBA2/N males (Charles River, Sulzfeld, Germany). They were housed in groups of six as described for the parental generation and were allowed to adapt for at least 20 days before testing started. All intruders were weighed prior to the first encounter to allow weight matching them to a respective resident. The heaviest intruder was determined for each cage and excluded from testing. These animals were used prior to the first RIT for instigation. Each instigation lasted 5 min, allowing to stimulate residents by visual and olfactory exposure to an intruder without the possibility of physical contact (de Almeida and Miczek, 2002; de Almeida et al., 2005). 3 min after the instigation ended the actual intruder was introduced into the resident’s home cage. The RIT was performed under red-light. In previous studies it has been reported that the initially observed level in aggression might not be representative of the true innate aggressive potential (Winslow and Miczek, 1984; Miczek et al., 2001; Caramaschi et al., 2008). Therefore, the RIT should be repeated several times, to allow animals to reach a stable level of offensive behavior. In the current study, the RIT was repeated four times with 24 h intervals between each encounter. For each encounter the resident was matched with an unfamiliar intruder. The test time added up to 5 min from the first attack bite (as observed online) or maximum 10 min. Encounters were filmed from a side-on view for subsequent behavioral analysis. An infrared-sensitive high-speed camera (The Imaging Source, Bremen, Germany) was used. Following each encounter, resident and intruder were examined for wounds. Recordings of the first and last of four encounters were analyzed in detail for all animals that were further screened for whole genome RNA expression and DNA methylation (*n* = 8–10 per group) using the Observer XT software (Noldus, Wageningen, Netherlands). Behaviors were scored in 5 categories, i.e., non-social, behavior independent of the intruder [rearing, digging, self-grooming and walking]; prosocial, affiliative behavior [sniffing, grooming, turning to, following, and looking toward (Miczek et al., 2001; Natarajan and Caramaschi, 2010; Koolhaas et al., 2013)], dominant, behavior to establish a clear hierarchy [grooming, mounting, and following, while the intruder showed clear signs of submission (Natarajan and Caramaschi, 2010; Wang et al., 2014)], threat, display of warning signs [tail rattling, sideway threatening, up-right posturing, boxing, aggressive grooming and fast following (Koolhaas et al., 2013)] and aggression [clinching, keeping down, biting, kicking and chasing (Koolhaas et al., 2013)]. The accumulated amount of time spent for each category was then analyzed relative to the total amount of time the animals spent in the encounter and is reported as percentage. Furthermore, to determine the quality of attacks, bite targets were scored in detail. To gain insight into rule breaking, we distinguished bites aimed at the back, throat, belly, face, paws and tail of the opponents. For further references, bites aimed at the back were declared social, as the targeted areas are non-vulnerable areas. Attacks aimed at the belly, face, paws throat and tail were determined unsocial, as they aimed at body parts, where vital organs are located or that are more prone to infection (Haller et al., 2001, 2005a; Blanchard et al., 2003; Koolhaas et al., 2013; Takahashi and Miczek, 2014). Besides bite targets, the occurrence of tail rattle, grooming, sniffing and mounting were scored.

### Measurement of Fecal Corticosterone Metabolites

To investigate functionality of the hypothalamic-pituitary-adrenal (HPA) axis, in a non-invasive manner, fecal boli, accumulated over the 7 day period between cage changes, were collected. This was done at three time-points throughout the experimental timeline: once before behavioral testing, once after EPM exposure and a last time at the day of sacrifice. Following sample collection, fecal boli were stored at -20°C, until further processing. Fecal corticosterone metabolites (FCMs) were extracted (50 mg powdered feces plus 1 ml 80% methanol) and, subsequently, measured with a 5α-pregnane-3ß,11ß,21-triol-20-one enzyme immunoassay (EIA) that was developed and successfully validated for mice (Touma et al., 2003, 2004).

### Epigenetic Regulation

For the investigation of epigenetic regulation both RNA and DNA were extracted from amygdala tissue of 8 animals per condition (48 animals in total), as described in more detail elsewhere (van den Hove et al., 2011; Schraut et al., 2014). RNA sequencing was performed by IGA Technologies (Udine, Italy) using the TrueSeq Stranded Total RNA kit and the Illumina HiSeq 2500 platform at a read-length of 125 bp with paired-end reads (60 million reads/sample). Mapping to the *mus musculus* GRCm38.p5 genome was performed by the Core Unit Systems Medicine at the University of Würzburg. To analyze DNA methylation, DNA capture-based sequencing was performed by Nxt-Dx (Ghent, Belgium). DNA was sheared by sonication followed by DNA capture using the methyl-CpG-binding domain (MBD) of human methyl-CpG binding protein 2 (MeCP2). Paired-end sequencing with 50 bp read-length (20 million reads/sample) was performed on the Illumina HiSeq4000 platform. Reads were mapped to the *mus musculus* GRCm38.p5 genome using Bowtie2 v2.1.0 software. After mapping, coverage peaks were generated using MACS 14 peak caller v1.4.2 (Zhang et al., 2008). Subsequently, peaks were aligned and sequencing reads within overlapping peak-sets were counted using the DiffBind R-package v2.0.9 (Stark and Brown, 2013). This resulted in count-tables of the methylated loci. Loci were annotated based on the first nearest feature, using ChIPpeakAnno v3.8.9 (Zhu et al., 2010) with the TxDb.Mmusculus.UCSC.mm10.ensGene v3.4.0. (2016) and ensemble-based EnsDb.Mmusculus.v75 v2.1.0 (Rainer, 2016) annotation packages. Sequencing data is deposited in the Gene Expression Omnibus database (identifier: GSE110330) and a more detailed description of RNA and DNA extraction and sequencing can be found in the Supplementary Methods (Data Sheet 1).

### Statistical Analysis

For statistical analysis of behavior, SPSS Statistics Version 23 or higher (IBM Deutschland GmbH, Ehningen, Germany) was used. Data was examined for normal distribution and outliers, using the Shapiro-Wilk test and boxplots. As a considerable number of factors did not meet assumptions for parametrical testing, Kruskal-Wallis tests, with grouping variables *Tph2* genotype, MS or experimental group (*Tph2*^∗^MS), were performed to test for main effects. Main effects were followed-up by Mann-Whitney U tests. For specific aggression parameters, such as target placement of attack bites, we performed Chi-Square test analysis for categorical data, indicating if more animals per grouping variable showed the investigated behavior. *P*-values were corrected for multiple comparisons per behavioral test, using the false discovery rate (FDR) online calculator^1^, correcting for measured parameters and applied statistical comparisons. Corrected *p* < 0.05 was considered significant. A table with all statistical comparisons is displayed in Supplementary Table 3 (Data Sheet 1). The fecal corticosterone metabolite profile was analyzed using repeated, multifactorial analysis of variance (ANOVA; time point, *Tph2* genotype and MS) with a Greenhouse-Geisser correction followed by Bonferroni-corrected *post hoc* analysis. To establish a normal distribution data was square root transformed.

For statistical analysis of RNA expression and DNA methylation, DESeq2 v1.14.1 (Love et al., 2014) was used in the free software environment R v3.3.2 (The R Foundation, Vienna, Austria), using default settings. Contrasts were calculated for *Tph2*^∗^MS interactions GE1 [(*Tph2*^+/-^ MS-*Tph2*^+/-^ C)-(*Tph2*^+/+^ MS-*Tph2*^+/+^ C)] and GE2 [(*Tph2*^-/-^ MS-*Tph2*^-/-^ C)-(*Tph2*^+/+^ MS-*Tph2*^+/+^ C)]. For RNA sequencing, this resulted in lists of differentially expressed genes (DEGs), comprising base mean of counts/gene, ratio on a log2 scale (log2 Fold Change; lg2FC), log fold change standard error, Wald statistic, *p*-value and adjusted *p*-value (Supplementary Table 4; Data Sheet 1). For this analysis, only genes with a base mean > 0 were taken into account and one gene (i.e., *Lars2*) was excluded due to an incomparably high base mean. A similar output was obtained for the analysis of the differentially methylated loci (DMLs) comprising, in addition to statistical results and peak loci, a list of annotated genes (Supplementary Table 5; Data Sheet 1). Pre-analysis quality control revealed two outlier samples, both of the *Tph2*^-/-^ MS condition, with remarkably lower counts, shifted normalized density and additional small peaks as well as an overall shifted peak in the density histogram of signal intensities (for quality control summary see Supplementary Figures 2, 3; Data Sheet 1). Those two samples were excluded from further analysis. Genes of either analysis were determined differential if they showed a nominal *p* < 0.01 and lg2FC > | 0.2|. Subsequently, the overlap of DEGs and DML-associated genes was determined and the relationship between overlapping DEG and DML raw counts as well as behavior was investigated using Spearman’s Rho (ρ). Correlation analysis was performed over all conditions. For statistically correlated DEGs and DMLs, differences in MS effects were investigated using Mann-Whitney U test. *P*-value correction was performed as described earlier. Furthermore, pathway enrichment analysis was conducted using the pathway analysis tool PathVisio (van Iersel et al., 2008; Kutmon et al., 2015), which operates based on the Wikipathways platform (Kelder et al., 2012; Kutmon et al., 2016). A detailed description and results of the pathway analysis are reported in Supplementary Methods and Supplementary Table 6 (Data Sheet 1).

## Results

### TPH2 Inactivation, Maternal Separation and Anxiety-Related Behaviors and Stress Response

**FIGURE 1:**
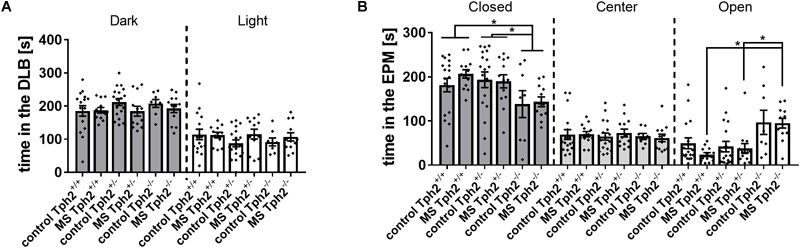
Anxiety-related behaviors in **(A)** the dark-light box (DLB) and **(B)** the elevated plus-maze (EPM): DLB performance was unaffected by both tryptophan hydroxylase 2 *(Tph2)* genotype and maternal separation (MS). In the EPM, full TPH2 inactivation increased the time [s] spent on open arms in offspring exposed to MS and decreased the time spent in closed arms, independent of MS. Bars represent group means ± standard errors (*n*/group = 8–18). ^∗^FDR corrected *p* < 0.050 (*Post hoc*: Mann-Whitney U).

In the DLB test, neither *Tph2* genotype, MS nor *Tph2*^∗^MS interaction affected the latency to enter the light compartment, distance moved (data not shown) or time spent (Figure 1A) in the dark or light compartment. In the EPM test, an effect of *Tph2*^∗^MS interaction was observed for time spent on the open arms [χ^2^(5) = 19.9, *p* = 0.008] and of *Tph2* genotype in the closed arms [χ^2^(2) = 10.2, *p* = 0.029], as depicted in Figure 1B. *Post hoc* analysis revealed that *Tph2*^-/-^ MS mice spent more time on the open arms than *Tph2*^+/-^ (*U* = 21.0, *p* = 0.010) and *Tph2*^+/+^ (*U* = 9.0, *p* = 0.004) MS mice. Furthermore, *Tph2*^-/-^ mice spent less time in the closed arms when compared to *Tph2*^+/-^ (*U* = 166.0, *p* = 0.014) and *Tph2*^+/+^ (*U* = 153.0, *p* = 0.013) mice. Similarly, the distance covered in the open arms was influenced by the *Tph2* genotype, independent of MS [χ^2^(5) = 26.6, *p* = 0.001]. Biallelic TPH2 inactivation increased the distance covered on the open arms, when compared to *Tph2*^+/-^ (C: *U* = 14.0, *p* = 0.010, MS: *U* = 24.0, *p* = 0.013) and *Tph2*^+/+^ (C: *U* = 19.0, *p* = 0.013, MS: *U* = 21.0, *p* = 0.010) mice. Also, entries into the open arms, time in the center and distance covered in the center were comparable between conditions and genotypes. In neither test did *Tph2* genotype, MS or *Tph2*^∗^MS interaction affect the total distance covered, while locomotion over 20 min in a non-aversive open-field was affected by the *Tph2* genotype (Supplementary Figure 1; Data Sheet 1; Weidner, 2018).

**FIGURE 2:**
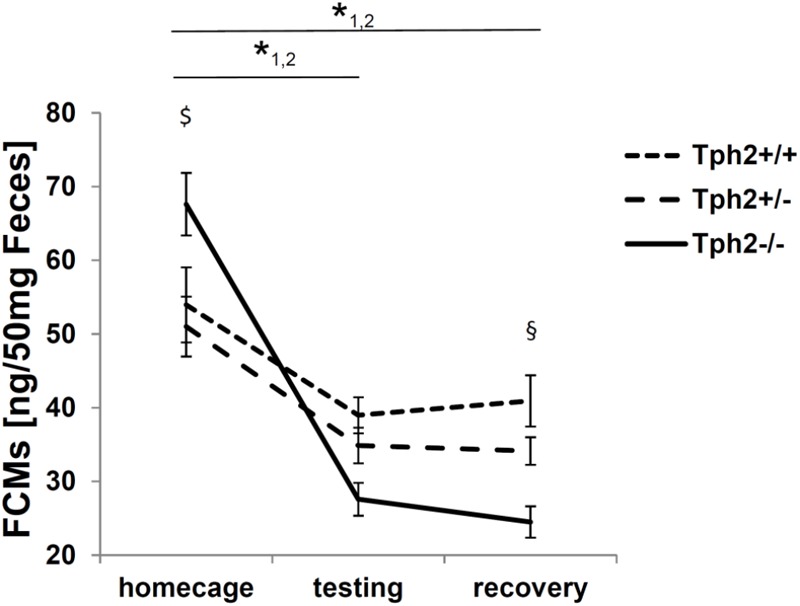
Fecal corticosterone metabolites (FCMs) in 50 mg feces. 1 *p* < 0.05 for home cage compared to testing and recovery in tryptophan hydroxylase 2 (TPH2)-deficient mice (*Tph2*^-/-^). 2 *p* < 0.05 for home cage compared to testing and recovery in Tph2^+/-^. $ *p* < 0.05 *Tph2*^-/-^ compared to *Tph2*^+/-^ under home cage conditions. § *p* < 0.05 *Tph2*^-/-^ compared to *Tph2*^+/+^ after recovery. Data points represent group means ± standard error (*n*/group = 8–12). Statistical analysis was performed using repeated measures ANOVA followed by Bonferroni corrected *t*-test.

The FCM profile recorded at three different time points (tp) throughout the experiment showed an effect of *Tph2* genotype over time [*F*(2.9,89.4) = 9.6, *p* = 2.03E-5] depicted in Figure 2. Over the three time-points (tp1, tp2 and tp3), the profiles of *Tph2*^-/-^ (tp1-tp2:p = 5.29E-13 tp1-tp3:p = 2.06E-15) and *Tph2*^+/-^ (tp1-tp2:p = 0.013 tp1-tp3:p = 0.009) mice’s FCM levels changed, while no significant change was observed in *Tph2*^+/+^ mice. At different time-points in particular *Tph2*^-/-^ mice showed a deviating FCM profile. At baseline *Tph2*^-/-^ mice’s feces showed increased FCM levels compared to the level in *Tph2*^+/-^ mice’s feces (*p* = 0.042) and at recovery *Tph2*^-/-^ mice’s feces showed decreased FCM levels compared to the level in *Tph2*^+/+^ mice feces (*p* = 0.002).

### TPH2 Inactivation, Maternal Separation and Territorial, Social Behaviors

**FIGURE 3:**
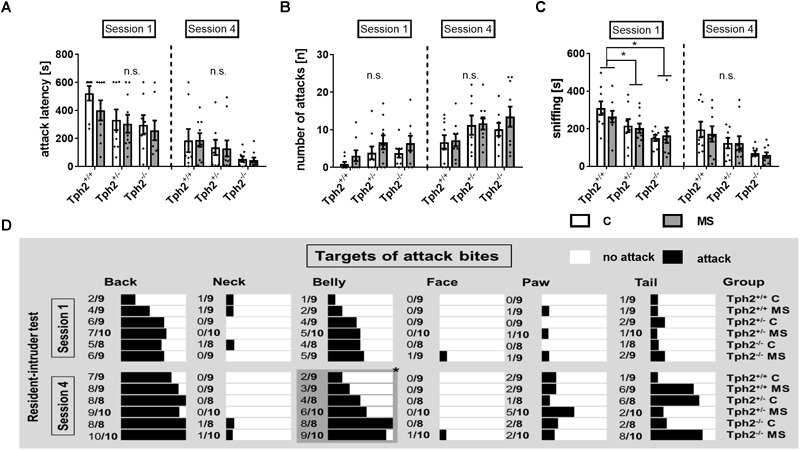
Social behaviors determined using repeated resident-intruder test (RIT). **(A)** Latency to attack [s] as well as **(B)** number of attacks [n] were not affected by the animals’ tryptophan hydroxylase 2 (*Tph2*) genotype, exposure to maternal separation (MS) or *Tph2*^∗^MS interaction during the first and fourth session of the RIT. **(C)** Sniffing [s] during the first session was decreased in mice carrying homo- and heterozygous genetic tryptophan hydroxylase 2 (TPH2) inactivation in both animals of the control (C) and MS condition. **(D)** During the fourth session targets of attack bites [nominal] of *Tph2*^-/-^ males were directed more toward vulnerable body parts, i.e., the belly, when compared to *Tph2*^+/-^ and *Tph2*^+/+^ males. **(A–C)** Bars represent group means ± standard errors (*n*/group = 8–10). ^∗^FDR-corrected *p* < 0.050 (*Post hoc*: Mann-Whitney U). **(D)** The relative number of animals per group by attack targets such as back and belly. The black bars represent the % of animals attacking the indicated target. ^∗^FDR-corrected *p* < 0.050 (Pearson chi-square test of categorical data, attack vs. no attack, n/group = 8–10).

For repeated RIT, the first and the last of four sessions were analyzed. In the first session, none of the classical parameters of RIT, like latency to attack (Figure 3A) or number of attacks (Figure 3B) showed any effect of 5-HT deficiency, MS or a *Tph2*^∗^MS interaction. Most prominently, *Tph2* genotype was found to affect the total amount of time mice were sniffing the intruder [χ^2^(2) = 15.8, *p* = 0.023; Figure 3C], with *Tph2*^-/-^ mice (*U* = 38.0, *p* = 4.42E-4) and *Tph2*^+/-^ mice (*U* = 92.0, *p* = 0.025) spending less time engaged in sniffing in comparison to *Tph2*^+/+^ mice.

During the fourth session, neither latency to attack (Figure 3A), nor number of attacks (Figure 3B) were affected by 5-HT deficiency, MS or a *Tph2*^∗^MS interaction A significant difference between genotypes, attacking the belly during the fourth session [χ(2) = 16.7, *p* = 0.023] was observed, with 95% of the *Tph2*^-/-^ mice targeting this body part at least once, while of *Tph2*^+/-^ mice 55% and of *Tph2*^+/+^ mice 28% attacked the belly (Figure 3D).

### TPH2 Inactivation, Maternal Separation and Epigenetic Regulation

**FIGURE 4:**
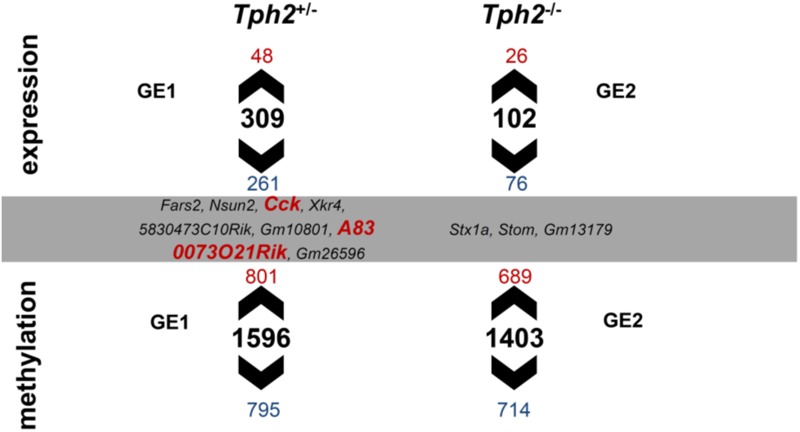
Overlap between differentially expressed genes (DEGs) and differentially methylated loci (DMLs) in the amygdala of mice that were exposed to maternal separation (MS) and are offspring of tryptophan hydroxylase 2-deficient (*Tph2*^+/-^) parents. For statistical analysis of RNA expression and DNA methylation, contrasts were calculated for *Tph2*^∗^MS interactions GE1 [(*Tph2*^+/-^ MS-*Tph2*^+/-^ C)-(*Tph2*^+/+^ MS-*Tph2*^+/+^ C)] and GE2 [(*Tph2*^-/-^ MS-*Tph2*^-/-^ C)-(*Tph2*^+/+^ MS-*Tph2*^+/+^ C)]. Per group 8 samples (total 48 samples were sequenced for either analysis). For DNA methylation data, pre-analysis quality control revealed two outlier samples, both of the *Tph2*^-/-^ MS condition, which were excluded from analysis. Genes of either analysis were determined differential if they showed a nominal *p* < 0.01 and lg2FC > | 0.2|. Fars2, phenylalanine-tRNA synthetase 2 (mitochondrial); Nsun2, NOL1/NOP2/Sun domain family member 2; Cck, cholecystokinin; Xkr4, X-linked Kx blood group related 4; 5830473C10Rik, RIKEN cDNA 5830473C10 gene; Gm10801, predicted gene 10801; A830073O21Rik, RIKEN cDNA A830073O21 gene; Gm26596, predicted gene 26596; Stx1a, syntaxin 1A (brain); Stom, stomatin; Gm13179, predicted gene 13179.

Gene expression and DNA methylation profiles were affected by the *Tph2*^∗^MS interactions GE1 [(*Tph2*^+/-^ MS-*Tph2*^+/-^ C)-(*Tph2*^+/+^ MS-*Tph2*^+/+^ C)] and GE2 [(*Tph2*^-/-^ MS-*Tph2*^-/-^ C)-(*Tph2*^+/+^ MS-*Tph2*^+/+^ C)] (Weidner, 2018). The results are displayed in Figure 4. For the interaction GE1 309 DEGs (DOWN 84.5%; UP 15.5%) and 1596 DMLs (DOWN 49.8%; UP 50.2%) were identified. The interaction GE2 was associated with 102 DEGs (DOWN 74.5%; UP 25.5%) and 1403 DMLs (DOWN 51.0%; UP 49.0%). Some DMLs were annotated to the same gene.

**FIGURE 5:**
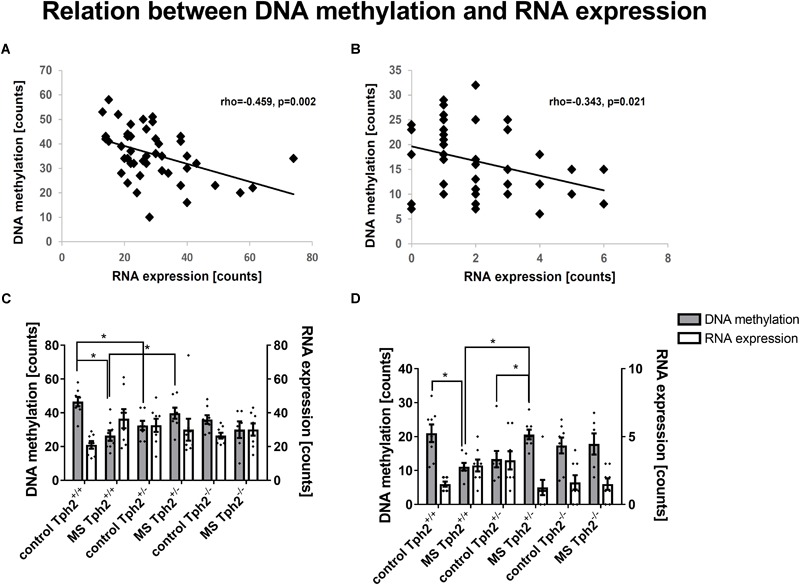
DNA methylation- and RNA expression-related correlations in amygdala. Amongst differentially expressed genes that are furthermore associated with differentially methylated loci, **(A)** the RIKEN DNA *A830073O21Rik* and **(B)** the neuropeptide cholecystokinin (*Cck*) are correlated amongst each other. Data based on sequencing counts of total RNA and methyl-CpG-binding domain (MBD) capture-based, enriched loci (*n*/group = 6–8). Correlation analysis was performed using the non-parametrical Spearman correlation (*n*/group = 5–8). Spearman correlation coefficient rho and *p*-value are reported. For both genes *A830073O21Rik* **(C)** and *Cck* **(D)** the comparison GE1 [(*Tph2*^+/-^ MS-*Tph2*^+/-^ C)-(*Tph2*^+/+^ MS-*Tph2*^+/+^ C)] with a significant difference between effect of neonatal, maternal separation (MS) compared to control in tryptophan hydroxylase 2 (*Tph2*) heterozygous mice and wildtype mice became apparent. *Post hoc* analysis revealed mainly group differences for *A830073O21Rik* and *Cck* methylation (*n*/group = 6–8). ^∗^FDR-corrected *p* < 0.050 (*Post hoc*: Mann-Whitney U test).

**FIGURE 6:**
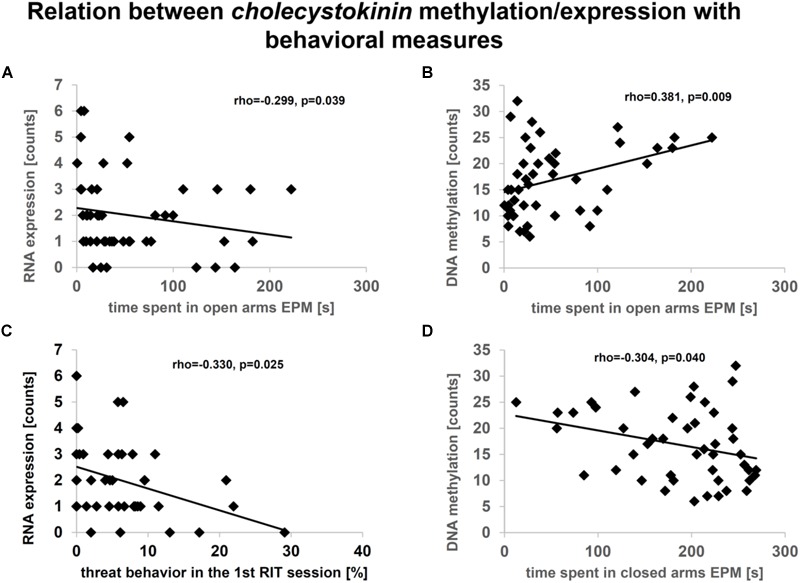
Relationship between cholecystokinin (Cck) expression **(A, C)** or methylation **(B, D)** in amygdala and behavior. Data based on sequencing counts of total RNA and methyl-CpG-binding domain (MBD) capture-based, enriched loci (*n*/group = 6–8). Analysis was performed using the non-parametrical Spearman correlation. Spearman correlation coefficient rho and *p*-value are reported. Correlation analysis was performed over all conditions.

Subsequently, the extent to which DML-associated genes and DEGs overlap was examined (Table 1). Amongst the genes identified in both profiles, only the predicted gene A830073O21Rik (ρ = -0.459, *p* = 0.002; Figure 5A) and cholecystokinin (*Cck*) (ρ = -0.343, *p* = 0.021; Figure 5B), both identified as significantly altered, dependent on the interaction between the *Tph2*^+/+^ and *Tph2*^+/-^ genotype with MS, showed a significant, negative relation between read counts of total RNA and MBD sequencing (Weidner, 2018). Based on these findings we analyzed the effects of *Tph2*^∗^MS interaction in detail, and found that, for *A830073O21Rik*, MS decreased the methylation in *Tph2*^+/+^ mice (*p* = 0.018) as shown in Figure 5C. In addition, *Tph2*^+/-^ mice of the control condition showed lower *A830073O21Rik* methylation compared to *Tph2*^+/+^ mice of the same condition (*p* = 0.036) and *Tph2*^+/-^ mice of the MS condition showed higher *A830073O21Rik* methylation compared to *Tph2*^+/+^ mice of the same condition (*p* = 0.039). For *A830073O21Rik* expression the *Tph2*^∗^MS interaction could not be attributed to single group comparisons, as none of the *post hoc* comparisons remained significant after FDR correction, suggesting more subtle effects compared to methylation (Figure 5C). For *Cck* we observed that MS decreased methylation in *Tph2*^+/+^ (*p* = 0.023) and increased methylation in *Tph2*^+/-^ mice (*p* = 0.039) as shown in Figure 5D. In addition, *Tph2*^+/-^ mice of the MS condition showed a higher *Cck* methylation compared to *Tph2*^+/+^ mice of the same condition (*p* = 0.009). For *Cck* expression, similar as observed for *A830073O21Rik* expression, the *Tph2*^∗^MS interaction could not be attributed to single group comparisons (Figure 5D). However, several correlations between the molecular and behavioral readings were observed. We found a positive correlation between *A830073O21Rik*-related DNA methylation and the distance moved in the light compartment of the DLB (ρ = 0.309, *p* = 0.034; data not shown). *Cck* expression positively related to levels of anxiety in the EPM (time open arm: ρ = -0.299, *p* = 0.039; Figure 6A) and *Cck*-related DNA methylation showed an opposing relationship with anxiety (time open arm: ρ = 0.381, *p* = 0.009; Figure 6B; closed arm: ρ = -0.304, *p* = 0.040; Figure 6D; distance on open arms: ρ = 0.403, *p* = 0.005; data not shown). Moreover, *Cck* expression was negatively correlated to threat behavior in the first RIT session (ρ = -0.330, *p* = 0.025; Figure 6C).

**Table 1:** Overlapping, differentially methylated loci (DMLs) and differentially expressed genes (DEGs).

Chr	start	end	symbol	Ensembl ID	biotype	Inside Feature	Bm	lg2FC	lfcSE	stat	*P*-value	*P* adj
**(A)**	**Contrast (GE1): [(*Tph2*^+/-^ MS-*Tph2*^+/-^ C)-(*Tph2*^+/+^ MS-*Tph2*^+/+^ C)]**					
	**DML**											
13	36365737	36366424	Fars2	ENSMUSG00000021420	protein_coding	inside	15.46	-0.74	0.21	-3.57	3.64E-04	0.96
13	69457460	69458618	Nsun2	ENSMUSG00000021595	protein_coding	upstream	37.21	-0.52	0.19	-2.79	5.34E-03	1.00
9	121477914	121478574	Cck	ENSMUSG00000032532	protein_coding	downstream	16.13	0.58	0.21	2.81	4.98E-03	1.00
1	3583857	3584649	Xkr4	ENSMUSG00000051951	protein_coding	inside	17.95	0.75	0.21	3.60	3.14E-04	0.96
5	90554052	90554958	5830473C10Rik	ENSMUSG00000070690	protein_coding	upstream	24.92	-0.69	0.19	-3.57	3.57E-04	0.96
2	98661728	98665716	Gm10801	ENSMUSG00000075015	protein_coding	includeFeature	96234.69	-0.44	0.12	-3.50	4.62E-04	1.00
7	73774794	73775963	A830073O21Rik	ENSMUSG00000091890	protein_coding	upstream	33.84	0.47	0.18	2.61	9.12E-03	1.00
10	112610921	112611527	Gm26596	ENSMUSG00000097185	protein_coding	upstream	15.17	-0.55	0.21	-2.64	8.40E-03	1.00
	**DEG**											
13	36117411	36537592	Fars2	ENSMUSG00000021420	protein_coding		10.02	-0.99	0.28	-3.59	3.37E-04	0.08
13	69533746	69635780	Nsun2	ENSMUSG00000021595	protein_coding		5.67	-0.84	0.26	-3.18	1.45E-03	0.14
9	121489825	121495689	Cck	ENSMUSG00000032532	protein_coding		1.96	-0.74	0.27	-2.74	6.10E-03	NA^∗^
1	3205901	3671498	Xkr4	ENSMUSG00000051951	protein_coding		3.54	-0.74	0.29	-2.59	9.62E-03	NA^∗^
5	90561107	90597871	5830473C10Rik	ENSMUSG00000070690	protein_coding		0.47	0.51	0.17	2.94	3.23E-03	NA^∗^
2	98662237	98664083	Gm10801	ENSMUSG00000075015	protein_coding		3.17	-0.86	0.26	-3.34	8.38E-04	NA^∗^
7	73737929	73741024	A830073O21Rik	ENSMUSG00000091890	TEC		28.22	-0.64	0.24	-2.63	8.47E-03	0.35
10	112928501	112931155	Gm26596	ENSMUSG00000097185	protein_coding		332.56	-0.48	0.19	-2.59	9.58E-03	0.37
**(B)**	**Contrast (GE2): [(*Tph*^-/-^ MS-*Tph2*^-/-^ C)-(*Tph2*^+/+^ MS-*Tph2*^+/+^ C)]**					
	**DML**											
5	135031000	135031630	Stx1a	ENSMUSG00000007207	protein_coding	inside	18.88	-0.56	0.21	-2.68	7.39E-03	1.00
2	35326274	35327079	Stom	ENSMUSG00000026880	protein_coding	inside	21.99	0.59	0.20	2.95	3.13E-03	1.00
2	4594896	4595707	Gm13179	ENSMUSG00000086018	antisense	upstream	16.98	-0.56	0.21	-2.65	8.13E-03	1.00
	**DEG**											
5	135023482	135051100	Stx1a	ENSMUSG00000007207	protein_coding		2.28	-0.71	0.27	-2.59	9.58E-03	1.00
2	35313986	35336976	Stom	ENSMUSG00000026880	protein_coding		0.37	-0.47	0.16	-2.91	3.67E-03	1.00
2	4586024	4587287	Gm13179	ENSMUSG00000086018	antisense		58.62	0.42	0.15	2.73	6.27E-03	1.00

*List of genes that had been affected by gene-by-environment interaction of tryptophan hydroxylase 2 (*Tph2*) genotype and maternal separation (MS), (GE1 [(*Tph2*^+/-^ MS-*Tph2*^+/-^ C)-(*Tph2*^+/+^ MS-*Tph2*^+/+^ C)] and GE2 [(*Tph2*^-/-^ MS-*Tph2*^-/-^ C)-(*Tph2*^+/+^ MS-*Tph2*^+/+^ C)]; reported under A and B, respectively). Data based on sequencing counts of total RNA and methyl-CpG-binding domain (MBD) capture-based, enriched loci (*n*/group = 6–8). Chr, chromosome; Bm, base mean; lg2FC, log2 fold change; lfcSE, log2 fold change standard error; stat, Wald-statistic; lincRNA, long intergenic non-coding RNA; lncRNA, long non-coding RNA; *p* adj, adjusted *p*-value; TEC, to be experimentally confirmed. Fars2, phenylalanine-tRNA synthetase 2 (mitochondrial); Nsun2, NOL1/NOP2/Sun domain family member 2; Cck, cholecystokinin; Xkr4, X-linked Kx blood group related 4; 5830473C10Rik, RIKEN cDNA 5830473C10 gene; Gm10801, predicted gene 10801; A830073O21Rik, RIKEN cDNA A830073O21 gene; Gm26596, predicted gene 26596; Stx1a, syntaxin 1A (brain); Stom, stomatin; Gm13179, predicted gene 13179. ^∗^Using the default settings of DESeq2; samples with certain criteria like low Bm are excluded from the analysis of *p* adj. As our analysis was based on nominal *p*-values; the standard analysis was not adapted.*

## Discussion

In the present study lifelong TPH2 inactivation and, consequently, depletion of brain 5-HT in *Tph2*^-/-^ mice induced substantial changes in socio-emotional behaviors, while heterozygous *Tph2*^+/-^ mice showed a more ambiguous, behavioral profile. Moreover, MS was shown to induce more subtle behavioral effects, which were dependent on the *Tph2* genotype. On the molecular level, we were able to relate various observed behavioral changes to expression and methylation of genes in the amygdala, potentially regulated through 5-HT and its interaction with MS.

In accordance with previously reported data (Gutknecht et al., 2015), we observed decreased anxiety in the EPM, as indicated by the time spent on the open arms, in *Tph2*^-/-^ mice. This was, however, dependent on the experience of MS during early life and *Tph2*^+/-^ and *Tph2*^+/+^ mice of the MS group showed similar levels of anxiety. Interestingly, though the time in closed arms as well as the distance on open arms was affected by *Tph2* genotype, independent of MS. Albeit effects in the EPM were very strong, no change was observable in the DLB. The discrepancy between these two approach-avoidance conflict-based anxiety tests might be owed to the nature of challenge and methodology. In the EPM, animals are placed into the center from which they can move into closed arms, which are perceived as safe, or onto open arms, which represent a threatening environment due to their elevated and more exposed nature (Lister, 1990). In the DLB animals are placed into the dark compartment, which is perceived as safe. From there, they are free to decide to move into the light compartment or remain in the dark compartment. Furthermore, in the EPM, appraisal of the situation is more complex due to the set-up of the maze (Casarrubea et al., 2013), while in the DLB, mice know the layout of the adjacent compartment after their first visit. Therefore, the observed deviation of behavior in the EPM seems to represent an altered appraisal in *Tph2*^-/-^ mice. Furthermore, our data suggests an altered exploratory activity based on MS in *Tph2*^-/-^ mice, as they spent more time and covered a greater distance on open arms when compared to their *Tph2*^+/+^ and *Tph2*^+/-^ littermates, while *Tph2*^-/-^ control mice only covered a greater distance, without spending more time on open arms compared to the respective *Tph2*^+/+^ and *Tph2*^+/-^ littermates. In a recent study, we reported a similar interaction of *Tph2* genotype, early adversity and acute challenge, in female mice (Auth et al., 2018). The genotype- and stressor-dependent behavioral effects in that study were associated with distinct patterns of brain activity, which furthermore depended on the nature of the acute challenge (i.e., the behavioral test). Findings by other groups, with regard to the effect of 5-HT deficiency on anxiety, were inconsistent (reviewed in Mosienko et al., 2015) and most likely to be accounted for by various factors such as the experimental read-out of the behavioral test used (Arabo et al., 2010) and the genetic background and life histories of investigated animals (Holmes et al., 2003). One hypothesis regarding an alternative read-out describes the time spent on the open arms as probing for a way out, describing increased flight-related behavior, rather than decreased anxiety. Regarding the genetic background, an extensive study, investigating 16 of the most frequently used laboratory mouse lines, discovered a variance of up to 78% between strains. Two of the strains with most opposing results regarding EPM behavior included C57BL6/J mice with an open-arm avoidance index of more than 90% and BALB/cJ mice with an index of 15% (Trullas and Skolnick, 1993). These two strains furthermore, showed opposing profiles when comparing aversive to non-aversive OF. Both strains showed low activity under dim light, with C57BL6 mice increasing their distance travelled under bright light, and BALB/c mice decreasing their distance travelled. Of note, BALB/c J mice carry a functional single nucleotide polymorphism in the *Tph2* gene, leading to a decreased TPH2 activity (Zhang et al., 2004; Kulikov et al., 2005; Bach et al., 2011).

Interestingly, monitoring FCM levels throughout the experiment revealed a genotype-dependent profile, which was totally unaffected by MS. We found in particular *Tph2*^-/-^ males deviated in their FCM profile compared to *Tph2*^+/-^ and *Tph2*^+/+^ mice. In contrast to previously reported, overall lower FCM levels in male *Tph2*^-/-^ mice, they showed a heightened FCM level at baseline, which might suggest a heightened activity of the HPA axis before behavioral testing in the current study. Following test exposure, *Tph2*^-/-^ offspring of the current study showed a more profound decrease in FCM levels compared to *Tph2*^+/+^ and *Tph2*^+/-^ mice. Interestingly, this profile seems to correspond with the activation profile of the basolateral amygdala, described before. In that study, Waider et al. (2017) observed a higher neural activation in *Tph2*^-/-^ males, under home cage conditions. This activation was abolished following exposure to a novel environment. Given the prominent role amygdala activity plays in HPA axis regulation (Herman et al., 2005), these findings might hint at a potential connection between the observed FCM levels and amygdala activity.

The observed, initially higher FCM levels might be explained by post-weaning environment (Hall, 1998). In the present study, males had been single-housed from weaning onward, which has been previously reported to alter the endocrine response in male mice (Bartolomucci et al., 2003). Furthermore, post-weaning social isolation was found to alter exploratory activity (Bartolomucci et al., 2003) as well as increase anxiety-related behaviors, which, only recently, was shown to be dependent on the corticotrophin releasing hormone (CRH) receptor 2 in DR (Bledsoe et al., 2011). The DR CRH receptor 2, has been associated with stress-induced TPH2 activity (Donner et al., 2016), suggesting a potential link with 5-HT deficiency. This might explain, why we observed an altered HPA axis activity in *Tph2*-deficient mice but not in their *Tph2*^+/+^ littermates. Moreover, 5-HT synapses have been identified on CRH-releasing cells in the PVN (Liposits et al., 1987) and 5-HT projections innervate relevant brain structures, such as the central amygdala (Asan et al., 2013) that are involved in regulating complex processes at the PVN (Herman and Cullinan, 1997; Van de Kar, 1997; Herman et al., 2005).

In the current study, in contrast to various other reports on early adversity, like MS, in rodents (Weaver et al., 2004; Weinstock, 2008; Wong et al., 2015), we were not able to find any effects of MS on HPA axis reactivity. One potential explanation for the lack of MS effects on the FCM profile might be that the MS paradigm, used in this study, was rather mild, even though similar paradigms reported strong effects. A moderating factor of MS in this regard might have been the maternal response to MS exposure (Own et al., 2013). The effect of maternal behavior and maternal stress level previously has been associated with the phenotype, observed in adult offspring (Meaney, 2001; de Souza et al., 2013; Murgatroyd et al., 2015). One mediating factor of maternal behavior is the genetic background. *Tph2*-deficient mice had previously been reported to exert altered maternal behavior (Angoa-Pérez and Kane, 2014) and altered stress reactivity (Gutknecht et al., 2015), both of which might have affected offspring development during MS. This is in line with the investigated behavioral parameters, which showed only subtle effects for MS, while most of the reported changes were induced by *Tph2* deficiency.

With regard to territorial, social behavior, *Tph2*-deficient animals had been reported to be significantly more aggressive in comparison to wildtype mice (Angoa-Pérez et al., 2012; Mosienko et al., 2012; Gutknecht et al., 2015). Although observable on a nominal level, none of the investigated aggression parameters showed a statistically significant increase in *Tph2*^-/-^ males in the current study. Furthermore, there was no effect of MS, supporting the previously discussed attenuating effect of maternal behavior also with regard to the spectrum of social behaviors. Interestingly though, the absolute time, *Tph2*-deficient males spent sniffing the intruder was significantly decreased during the first encounter, suggesting altered prosocial behavior in mice with lifelong 5-HT deficiency. This finds support in the fact that several prosocial parameters measured in this study approach significance with an FDR-corrected *p* < 0.10. The observed anti-social effect was particularly strong in *Tph2*^-/-^ males. Comparable results were reported earlier (Kane et al., 2012; Waider et al., 2017), where *Tph2*^-/-^ mice displayed e.g., decreased social interaction as well as less preference for socially relevant odors such as urine and, while showing a comparable habituation, failed dishabituation after the introduction of a novel social target. Testing general olfactory capacities in those animals did not reveal any defects in TPH2-deficient mice (Kane et al., 2012). Of note, social contact was found to activate raphe neurons, in rats, supporting the idea of 5-HT involvement in normal social behaviors (Haller et al., 2005b). A lack of 5-HT response to the initial activation of raphe neurons, thus, might impair the animals’ appraisal and, consequently, affect the behavioral reaction to the intruder. In line with this, we observed deviations from normal behavioral patterns regarding the aggressive interaction in 5-HT-deficient mice. We found that more *Tph2*^-/-^ mice attacked the belly of intruders during the fourth session of the RIT. The belly is a vulnerable body part and attacks toward it are usually avoided in offensive, aggressive encounters (Litvin et al., 2007). Thus, *Tph2*^-/-^ mice seem to be less restricted by the social convention of attack placements and display rule-breaking behavior. Interestingly, adrenalectomy induced a similar increase in attacks aimed at vulnerable targets in rats (Haller et al., 2001) and abolished the positive relationship between attack bites at non-vulnerable regions and 5-HT neuron activity in DR (Haller et al., 2005b). This is emphasizing the close interaction between 5-HT transmission and stress response.

Taken together, results of the behavioral screening suggest two major conclusions. Firstly, *Tph2*^-/-^ mice might perceive a stressful stimulus differently when compared to their *Tph2*^+/+^ counterparts. Under aversive conditions 5-HT deficiency altered the approach toward potentially aversive cues. This might be mediated via an altered regulation of the stress response. An alternate explanation for the abnormal behavior in the EPM might be a dysregulation of 5-HT-dependent signaling involved in the regulation of anxiety- and panic-related behaviors (Paul et al., 2014). As described in the Deakin and Graeff hypothesis 5-HT is necessary to suppress the active motor reaction emerging from the PAG. Thus, lack of 5-HT, might be driving behavior toward a more activity-oriented coping strategy (Paul et al., 2014; Auth et al., 2018). However, in the current model it is not possible to determine if the observed behavioral consequences arise from an acute deficit in 5-HT, as would be suggested by the Deakin and Graeff hypothesis or if compensatory, developmental mechanisms are taking effect. Furthermore, based on our observation that exaggerated behavior was mostly observed in the context of aversive stimuli, a combination of altered appraisal and behavioral disinhibition might explain the observed behavioral phenotype. Notably, *Tph2*^+/-^ mice displayed an inconclusive behavioral profile. This is most likely due to compensatory mechanisms involving decreased degradation of 5-HT (Márquez et al., 2013; Mosienko et al., 2014). *Tph2*^+/-^ mice had been observed to show reductions of only 10–20% in 5-HT levels (Mosienko et al., 2012). This disproportional reduction in brain 5-HT is suggested to be a consequence of compensatory processes, involving e.g., decreased activity of MAO-A (Mosienko et al., 2014). Moreover, the behavioral distinction between *Tph2*^+/-^ and *Tph2*^-/-^ mice, or, dependent on the interrogated task, *Tph2*^+/+^ mice might be dependent on the involved brain circuitries, of which each might affect unique features. Therefore, *Tph2*^+/-^ offspring might show phenotypes, related to *Tph2*^-/-^ offspring or *Tph2*^+/+^ offspring, dependent on the task and experienced aversiveness.

Secondly, MS exerted only indirect effects by reinforcing *Tph2* genotype-dependent, behavioral effects. Based on previous work, we did expect an effect of MS on anxiety- and aggression-related behaviors (Veenema et al., 2006, 2007). One potential explanation for the lack of consistent MS effects might be based on the involvement of several modulating factors. As discussed earlier, a moderating factor of MS in this regard might have been the maternal response to MS exposure (Own et al., 2013). The molecular basis of this phenomenon of less effect of adverse factors has been investigated in manifold experiments and a number of behavioral, physiological and molecular factors were identified as relevant mediators of individual susceptibility to stress (Jakob et al., 2014; Anacker et al., 2016; Han and Nestler, 2017). As one important factor of individual stress susceptibility epigenetic modifications such as DNA methylation have been identified (Dudley et al., 2011). As set out in the introduction epigenetic modifications are affected by environmental factors and the animal’s genetic make-up e.g., with regard to functional 5-HT system components.

To investigate the molecular underpinnings of *Tph2* function-dependent effects of MS, whole genome RNA expression and DNA methylation profiling was performed in tissue homogenate of amygdala, which comprises a multilayered assembly of subregions and neuronal types that are involved in the regulation of complex behaviors in a highly specific manner (Asan et al., 2013). The amygdala is extensively innervated by serotonergic terminals (Asan et al., 2013) and has been reported as one of the structural entities regulating coping-related behavioral reaction to environmental challenges (Hale et al., 2008a,b, 2010; Tovote et al., 2016). Recently, altered neural activity in the basolateral amygdala of male *Tph2*^-/-^ mice compared to their *Tph2*^+/-^ littermates under home cage conditions was reported (Waider et al., 2017), while *Tph2*^+/-^ and *Tph2*^+/+^ mice did not differ in this respect. Electrophysiological investigation of the excitability of amygdala neurons revealed altered amygdala reactivity in *Tph2*^-/-^ and *Tph2*^+/-^ males. Priming the amygdala using the stress-related neuropeptide urocortin 1 resulted not only in increased social anxiety, but was also linked to *Tph2* expression changes (Donner et al., 2012). Overall, and given the role amygdala activity plays in stress response regulation (Herman et al., 2005), it represents a relevant hub of 5-HT signaling and early-life programming. However, characterization of other regions involved in anxiety and social behaviors is warranted.

Within the amygdala, we found that the number of DEGs for gene-by-environment interactions differed notably, dependent on the *Tph2* genotype. DNA methylation changes were found to be relatively equal across comparisons. This discrepancy between methylation and expression changes suggests a *Tph*2-dependent effect on early-life programming by MS, partially independent of DNA methylation. Notably, only a fraction of the DEGs were found to be associated with DMLs, and of the overlapping genes only two showed a statistically significant correlation between expression and DNA methylation. This relatively low rate of DML-associated genes, overlapping with DEGs and the lack of significant correlation between expression and methylation of genes is in accordance with findings in other studies (Schraut et al., 2014) and most likely explained by the highly complex interaction of a multitude of epigenetic and structural factors, regulating gene expression (Jenuwein and Allis, 2001; Narlikar et al., 2002; Barski et al., 2007; Garske et al., 2010; Kratz et al., 2010; Fuchs et al., 2011). Furthermore, it has to be considered, that the method of MBD capture is not specific for cytosine methylation, but to a certain extent will also capture cytosine hydroxymethylation (Du et al., 2015). However, the binding capacity for this cytosine modification is several magnitudes less compared to their affinity for methylated cytosines at CpG sites. Lastly, it has to be mentioned that the investigated tissue has been taken after the animals experienced a variety of behavioral tests. Therefore, it might be possible, that several effects of *Tph2*^∗^MS interaction have been compensated or reinforced by further life experiences associated with behavioral testing, which has previously been shown (van den Hove et al., 2014). Nevertheless, designed to model aversive environment in humans, the inclusion of further life experiences is naturalistic.

Despite these limitations, we uncovered an interesting candidate-gene, *Cck*, to be involved in mediating the effects of early adversity dependent on 5-HT. As one of two genes showing an associated expression and methylation for the interaction of the *Tph2*^+/-^ genotype and MS, *Cck* was furthermore, extensively associated with observed behaviors. For example, lower expression of *Cck* was related to more relative time displaying threat in the first session of the RIT. Furthermore, lower expression and higher methylation of *Cck* were associated with lower levels of anxiety. In particular with regard to this association, we observed an interaction across the levels of DNA methylation, RNA expression, and behavior. More specifically, *Cck* methylation was found to be decreased and its expression increased by MS in *Tph2*^+/+^ offspring, while in *Tph2*^+/-^ offspring MS had an opposing effect, leading to an increase in *Cck* methylation and a decrease in expression. In *Tph2*^-/-^ offspring, MS had no effect on either *Cck* methylation, or expression and showed constantly low expression and high methylation levels. This might indicate gene-by-environment-dependent epigenetic programming and altered susceptibility to MS in *Tph2*^-/-^ offspring.

CCK is a peptide that was originally identified in the gut. However, it appears in greater amounts in the brain than in the periphery and is one of the most abundant neuropeptides (Innis et al., 1979; Crawley, 1985; Moran and Schwartz, 1994). Effects of CCK are mediated by CCK1 and CCK2 receptors (Moran et al., 1986). The CCK2 receptor is mainly found in the brain and shown to be involved in emotion regulation (Hughes et al., 1990; Chen et al., 2006). A study in rats, investigating CCK system functioning following MS, showed an increase in sensitivity toward CCK-4 injections in animals that were subjected to a separation paradigm (Greisen et al., 2005), supporting the idea of MS-induced epigenetic regulation. Of note, adrenalectomized rats, known to display increased abnormal aggressive behavior, showed a lower expression of CCK and decreased activation of CCK-expressing neurons in the prefrontal cortex (Haller et al., 2005a). Decreased CCK-positive neuron activation was furthermore related to the increased incident of vulnerable attacks, in line with the observed behavior and low *Cck* expression profile in *Tph2*^-/-^ mice. In general, CCK has been shown to interact with the 5-HT system. In rats, administration of the selective 5-HT1a receptor antagonists (+)WAY100135 and WAY100635 resulted in an attenuation of aversion-related behaviors that were induced by CCK-8 injection (Bickerdike et al., 1995) while CCK_B_ receptor antagonist reversed reduced exploratory activity induced by selective serotonin reuptake inhibitor (Kõks et al., 1999). Direct 5-HT administration was shown to enhance CCK release via activation of 5-HT3 receptors (Raiteri et al., 1993). Taken together these results suggest a positive association between 5-HT and CCK, which is in line with the observations in the current study, where mice, completely depleted of 5-HT showed low levels of *Cck* expression independent of MS. In limbic regions, such as the hypothalamus and amygdala, converged effects of 5-HT and CCK occur at a subset of neurons, allowing for direct modulatory interaction of these signaling molecules (Zippel et al., 1999; Asan et al., 2013). Moreover, CCK is particularly associated to anxiety- and panic-related behaviors across species (Harro et al., 1993). This has been further discussed in light of the 5-HT hypothesis of defense (Graeff et al., 2015), where the authors conclude that CCK neurotransmission is positively associated with escape-related behaviors, such as the escape response in the elevated T-maze, while 5-HT neurotransmission on the contrary is negatively associated with these behaviors. This is in contrast to the positive relationship observed between CCK and 5-HT, discussed earlier and also observed in the current study. Potential explanations for this discrepancy are brain region specificity, most likely dependent on the available 5-HT receptors and further modulatory factors. A clear interaction between CCK and 5-HT seems to be likely, whereby 5-HT might serve as modulatory interface between environment and CCK expression and consequently effect by affecting epigenetic regulation. All in all, the involvement of *Cck* in the observed 5-HT-dependent behavioral aberrations seems to be likely and altered *Cck* regulation by MS might represent a potential, 5-HT-dependent mechanism of early-life adversity.

Next to *Cck*, the RIKEN cDNA *A830073O21* was found to be modulated by the interaction of 5-HT depletion and MS in a similar pattern as observed for *Cck* expression and methylation. It is an unclassified gene located on chromosome 7 that was suggested to be a mitochondrial protein in a combined mass spectrometry, GFP tagging, and machine learning approach resulting in 1058 gene assembly associated with mitochondria called MitoCarta (Pagliarini et al., 2008), but no longer appeared in the MitoCarta 2.0 (Calvo et al., 2016). Further work toward elucidating its function is thus warranted. Its comparably high expression (amongst the top 10%) in the amygdala and the equally high susceptibility to gene-by-environment-dependent effects suggest a role in 5-HT-dependent response to environmental cues. This finds further support by a predicted interaction with in total 164 microRNAs^2^.

Taken together, our results highlight epigenetic *Cck* regulation in the amygdala by early-life adversity as potential mechanism involved in anxiety-like and aberrant social behaviors of a lifelong deficiency in 5-HT synthesis.

## Data Availability

The datasets GSE110330 generated for this study can be found in NCBI GEO https://www.ncbi.nlm.nih.gov/geo/query/acc.cgi?acc=GSE110330. Other relevant data is provided as Supplementary Material.

## Ethics Statement

This study was carried out in accordance with the recommendations of the European Parliament and Council Directive (2010/63/EU). The protocol was approved by local authorities (Government of Lower Franconia, Würzburg: 55.2-2531.01- 57/12).

## Author Contributions

K-PL, DvdH, and MW conceived and designed the study. MW, FM, LDG, and RP acquired the data. RL, LE, and KF performed bioinformatics processing and analysis of sequencing data. MW wrote the first draft of the manuscript. MW, DvdH, K-PL, JW, JG, RL, LE, FM, AS-B, SP, TS, RP, and HS contributed to the interpretation of the data, preparation of the figures, writing and/or revising of the manuscript.

## Conflict of Interest Statement

The authors declare that the research was conducted in the absence of any commercial or financial relationships that could be construed as a potential conflict of interest.

## Footnotes

1 https://www.sdmproject.com/utilities/?show=FDR

2 http://www.informatics.jax.org/interaction/explorer?markerIDs=MGI:2443692, as consulted online on 11.04.2019
