# Supplementary material for: Identification of Cholecystokinin by Genome-Wide Profiling as Potential Mediator of Serotonin-Dependent Behavioral Effects of Maternal Separation in the Amygdala
Source: Front Neurosci. 2019 May 10;13:460. doi: 10.3389/fnins.2019.00460 (PMC6524554; doi:10.3389/fnins.2019.00460)
Supplement: Supplementary file 4 [file Data_Sheet_1.pdf]

Supplementary Material

1 Supplementary Methods

1.1 Behavioral testing

Behavioral tests were performed, starting with the dark-light box (DLB) test, followed by open-field (OF) test and elevated plus maze (EPM) and as last test the repeated resident-intruder test (RIT). The order, in which animals were tested, was randomized over genotype and treatment condition, in a way, that a nearly equal number of animals of each experimental group were tested per day, to avoid bias. For all tests, unless stated otherwise, mice were tracked from above, using infrared light from below the respective apparatus to increase contrast. Trials were recorded from above, using an infrared-sensitive CCD camera. Later on, behavioral analysis was performed using VideoMot2 tracking software (TSE Systems, Bad Homburg, Germany). In-between trials, the respective apparatus was cleaned with Terralin liquid (Schülke, Norderstedt, Germany).

1.1.1 Dark-Light box

The DLB test is an approach-avoidance conflict paradigm and was used to determine the innate level of avoidance behavior (Crawley & Goodwin, 1980; Onaivi & Martin, 1989). The DLB apparatus is an arena made from opaque, white, acrylic glass, semi-permeable to infrared light, with an edge length of 50 cm. This arena is divided into a light compartment (34 cm x 50 cm x 40 cm) with on average 115 lux in the center and 80 lux in the corners and a dark compartment (16 cm x 50 cm x 40 cm) with 0.1 lux. The two compartments are connected through an exit hole and animals can move freely between compartments. Each tested animal was placed into the dark compartment, facing the front right corner, and was allowed to explore the arena freely for 5 min (Waider *et al*, 2017). Subsequently, distance moved and time, spent in the dark and in the light compartment as well as the latency to enter the light compartment and total distance moved were analyzed.

1.1.2 Open-field

The OF test was used to determine exploratory behavior and locomotion in a novel, environment. The OF apparatus is an open arena made from infrared semi-permeable, acrylic glass with an edge length of 50 cm and a wall height of 40 cm. The test was performed under red-light (Post *et al*, 2011). The tested animal was placed into the arena facing the front right corner and was allowed to explore the arena freely for 20 min. Subsequently, distance and time in the center and global distance moved were analyzed (Supplementary Figure 1).

1.1.3 Elevated plus-maze

The EPM test is a conflict anxiety test, testing avoidance behaviors, similar to the DLB test. The EPM apparatus is a plus-shaped acrylic glass construct (TSE Systems, Inc., Bad Homburg, Germany) made from black opaque PERSPEX XT, semi-permeable to infrared light, with two opposing closed arms (30 cm × 5 cm), comprised by 15 cm high walls, and two opposing open arms without walls (30 cm × 5 cm, with 0.5 cm wide boundaries elevated 0.2 cm). The four arms meet in the center to form a square of 5 cm × 5 cm. The maze was raised 62.5 cm above ground. On the open arms, the brightness measured was on average 70 lux, in the center 20 lux, and in closed arms 1 lux. Each animal was

placed in the center facing an open arm and allowed to explore the maze for 5 min (Post *et al*, 2011; Gutknecht *et al*, 2015). Subsequently, distance and time on open arms, in closed arms and the center, as well as the number of entries onto the open arms were analyzed (Lister, 1987).

1.2 RNA and MBD based sequencing

For the whole genome approaches, both DNA and RNA were extracted from amygdala tissue. Frozen brains were semi-thawed on a cooling plate (-6°C) and the amygdala was rapidly dissected using a stereo microscope (Olympus, Hamburg, Germany). Before extracting nucleic acids, the tissue of left and right amygdala was powderized on dry ice, using a precooled metal mortar, blended and split into two homogenous portions, of which one portion was used to investigate RNA expression, the other to investigate DNA methylation.

1.2.1 Total RNA sequencing

Extraction of RNA was performed, using a combination of the classic phenol-chloroform method and the column-based protocol of the commercial miRNeasy Mini kit (Qiagen, Hilden, Germany) for fatty tissue. The frozen samples were homogenized in QIAzol lysis reagent with a precooled stainless steel bead (Qiagen), using the TissueLyzer (Qiagen) at 20 Hz and 4°C, for 60 s. Subsequently, homogenates were incubated for 5 min at room temperature (RT), mixed with 60 µl of chloroform (Roth, Karlsruhe, Germany) and incubated for another 10 min on ice before the aqueous phase was recovered by phase separation and mixed with 1.5 volumes ethanol (95-100%). Then samples were transferred to miRNeasy Mini columns and processed according to kit instructions. To minimize the risk for genomic DNA contamination the samples were incubated with DNase (Qiagen). Extracted RNA was finally stored at -80°C. RNA quality was assessed before further processing using the Experion capillary electrophoresis (Biorad, München, Germany) according to manufacturer's instructions. Only samples with Experion RNA quality indicator (RQI) values between 8.0 and 9.8 were considered for further analysis. In addition, RNA concentrations were determined using Nanodrop Microvolume Spectrophotometer (Thermo Fisher Scientific, Waltham, MA USA).

For next-generation sequencing, 1 µg RNA was shipped to IGA Technologies (Udine, Italy) for further processing. Library preparation was performed using the TruSeq Stranded Total RNA Library Prep Kit (Illumina, San Diego, CA, US) that captures coding RNA and multiple forms of non-coding RNA, with a total input of 300 ng of RNA, as measured with Qubit 2.0 Fluorometer (Invitrogen, Carlsbad, CA) following manufacturer's recommendations. The sequencing was performed using the Illumina HiSeq 2500 platform at a read-length of 125 bp with paired-end reads, yielding 60 million reads/sample. CASVA v1.8.2 illumina pipeline was used for format conversion and demultiplexing. Mapping was performed by the Core Unit Systems Medicine at the University of Würzburg, where reads were mapped to the *mus musculus* GRCm38.p5 genome using STAR (Dobin *et al*, 2013). Subsequently, the reads per position were determined using HTSeq (Anders *et al*, 2015) resulting in count-tables.

1.2.2 DNA methylation enrichment-based sequencing

Extraction of genomic DNA was performed using a standard phenol/chloroform/isoamyl alcohol extraction protocol. The frozen samples were homogenized in 0.5 % SDS extraction buffer with a precooled stainless-steel bead (Qiagen) using the TissueLyzer (Qiagen) at 20 Hz and 4°C, for 60 s. Subsequently, samples were incubated with 500 µg proteinase K (Applichem, Darmstadt, Germany) at 55°C for at least 3 h followed by 1 h incubation with 500 µg RNase A (Roche, Basel, Switzerland)

at 37 °C. Subsequent DNA isolation through phase separation and DNA precipitation from the aqueous phase were performed as described elsewhere (Schraut *et al*, 2014). DNA was stored at -80°C for further use. DNA concentrations were determined using the Qubit fluorometer (Thermo Fisher Scientific) with the Qubit high sensitivity kit for double-stranded DNA (Thermo Fisher Scientific).

For next-generation sequencing, 300 ng DNA were shipped to Nxt-Dx (Ghent, Belgium) for further processing. Briefly, DNA was sheared by sonication followed by DNA capture using methyl binding domain (MBD) of human methyl-CpG binding protein 2 (MeCP2). Subsequently, library preparation was performed followed by multiplexed paired-end sequencing with a read-length of 50 bp and an approximate output of 20 million reads/sample on the Illumina HiSeq4000 platform. Following data demultiplexing, clean-up and quality control, which was performed using FastQC quality control v0.10.1, reads were mapped to the *mus musculus* GRCm38.p5 genome using Bowtie2 v2.1.0 software in "end-to-end & sensitive-mode". After mapping, coverage peaks were generated using MACS 14 peak caller v1.4.2 (Zhang *et al*, 2008). Subsequently, peaks were aligned and sequencing reads within overlapping peak sets were counted using the DiffBind R-package v2.0.9 (Stark & Brown, 2013). This resulted in count-tables of the methylated loci. Genes were annotated based on the first nearest feature, using ChIPpeakAnno v3.8.9 (Zhu *et al*, 2010) with the TxDb.Mmusculus.UCSC.mm10.ensGene v3.4.0. (2016) and EnsDb.Mmusculus.v75 v2.1.0 (Maintainer: J Rainer 2016) annotation packages. A detailed dry lab report of Nxt-Dx can be provided upon request.

1.3 Pathway analysis

In addition to candidate-based analysis, pathway enrichment analysis was conducted using the pathway analysis tool PathVisio (Kutmon *et al*, 2015; van Iersel *et al*, 2008), which operates based on the Wikipathways platform (Kelder *et al*, 2012; Kutmon *et al*, 2016). For analysis, the output of both RNA and MBD sequencing were sorted based on the p-value, from lowest to highest p-value. Unique gene names annotated to the top 500 genes/loci of these lists were used as input. Of the top 10 pathways, only pathways with a z-score > 2 and an enrichment of more than 3 genes were considered as enriched. To determine affected genes, the online tool EnrichR (Chen *et al*, 2013; Kuleshov *et al*, 2016) was used. Results are displayed in Supplementary Table 6.

1.4 References

Anders S, Pyl PT & Huber W (2015) HTSeq-A Python framework to work with high-throughput sequencing data. *Bioinformatics* **31:** 166–169

Chen EY, Tan CM, Kou Y, Duan Q, Wang Z, Meirelles G, Clark NR & Ma'ayan A (2013) Enrichr: interactive and collaborative HTML5 gene list enrichment analysis tool. *BMC Bioinformatics* **14:** 128 Available at: http://bmcbioinformatics.biomedcentral.com/articles/10.1186/1471-2105-14-128

Crawley J & Goodwin FK (1980) Preliminary report of a simple animal behavior model for the anxiolytic effects of benzodiazepines. *Pharmacol. Biochem. Behav.* **13:** 167–170

Dobin A, Davis CA, Schlesinger F, Drenkow J, Zaleski C, Jha S, Batut P, Chaisson M & Gingeras TR (2013) STAR: ultrafast universal RNA -seq aligner. *Bioinformatics***:** 1–7

Gutknecht L, Popp S, Waider J, Sommerlandt FMJ, Göppner C, Post A, Reif A, Van Den Hove D, Strekalova T, Schmitt A, Colaço MBN, Sommer C, Palme R & Lesch KP (2015) Interaction of brain 5-HT synthesis deficiency, chronic stress and sex differentially impact emotional behavior in Tph2 knockout mice. *Psychopharmacology (Berl).* **232:** 2429–2441

van Iersel MP, Kelder T, Pico AR, Hanspers K, Coort S, Conklin BR & Evelo C (2008) Presenting and exploring biological pathways with PathVisio. *BMC Bioinformatics* **9:** 399

Kelder T, Van Iersel MP, Hanspers K, Kutmon M, Conklin BR, Evelo CT & Pico AR (2012) WikiPathways: Building research communities on biological pathways. *Nucleic Acids Res.* **40:** 1301–1307

Kuleshov M V., Jones MR, Rouillard AD, Fernandez NF, Duan Q, Wang Z, Koplev S, Jenkins SL, Jagodnik KM, Lachmann A, McDermott MG, Monteiro CD, Gundersen GW & Ma'ayan A (2016) Enrichr: a comprehensive gene set enrichment analysis web server 2016 update. *Nucleic Acids Res.* **44:** W90–W97

Kutmon M, van Iersel MP, Bohler A, Kelder T, Nunes N, Pico AR & Evelo CT (2015) PathVisio 3: An Extendable Pathway Analysis Toolbox. *PLoS Comput. Biol.* **11:** 1–13

Kutmon M, Riutta A, Nunes N, Hanspers K, Willighagen EL, Bohler A, Mélius J, Waagmeester A, Sinha SR, Miller R, Coort SL, Cirillo E, Smeets B, Evelo CT & Pico AR (2016) WikiPathways: Capturing the full diversity of pathway knowledge. *Nucleic Acids Res.* **44:** D488–D494

Lister RG (1987) The use of a plus-maze to measure anxiety in the mouse. *Psychopharmacology (Berl).* **92:** 180–185

Onaivi ES & Martin BR (1989) Neuropharmacological and physiological validation of a computer-controlled two-compartment black and white box for the assessment of anxiety. *Prog. Neuropsychopharmacol. Biol. Psychiatry* **13:** 963–976

Post AM, Weyers P, Holzer P, Painsipp E, Pauli P, Wultsch T, Reif A & Lesch KP (2011) Gene-environment interaction influences anxiety-like behavior in ethologically based mouse models. *Behav. Brain Res.* **218:** 99–105 Available at: http://dx.doi.org/10.1016/j.bbr.2010.11.031

Schraut KG, Jakob SB, Weidner MT, Schmitt a G, Scholz CJ, Strekalova T, El Hajj N, Eijssen LMT, Domschke K, Reif a, Haaf T, Ortega G, Steinbusch HWM, Lesch KP & Van den Hove DL (2014) Prenatal stress-induced programming of genome-wide promoter DNA methylation in 5-HTT-deficient mice. *Transl. Psychiatry* **4:** e473 Available at: http://www.nature.com/doifinder/10.1038/tp.2014.107

Stark R & Brown G (2013) DiffBind : differential binding analysis of ChIP-Seq peak data DiffBind works primarily with peaksets , which are sets of genomic intervals representing candidate. **:** 1–40

Waider J, Popp S, Lange MD, Kern R, Kolter JF, Kobler J, Donner NC, Lowe KR, Malzbender JH, Brazell CJ, Arnold MR, Aboagye B, Schmitt-Böhrer A, Lowry CA, Pape HC & Lesch KP (2017) Genetically driven brain serotonin deficiency facilitates panic-like escape behavior in mice. *Transl. Psychiatry* **7:** e1246 Available at:

http://www.nature.com/doifinder/10.1038/tp.2017.209

Zhang Y, Liu T, Meyer CA, Eeckhoute J, Johnson DS, Bernstein BE, Nusbaum C, Myers RM, Brown M & Li W (2008) Model-based analysis of ChIP-Seq (MACS). *Genome Biol* **9:**

Zhu LJ, Gazin C, Lawson ND, Pagès H, Lin SM, Lapointe DS & Green MR (2010) ChIPpeakAnno: A Bioconductor package to annotate ChIP-seq and ChIP-chip data. *BMC Bioinformatics* **11:**

2 Supplementary Figures and Tables

2.1 Physiology

Supplementary Table 1 . Physiological effects of maternal separation on dams and litters. 17 tryptophan hydroxylase 2 deficient (*Tph2*$^{+/-}$) dams and their respective litters were subjected to maternal separation (MS). The same number of dams and litters were left undisturbed except for weighing at postnatal day (P)5, 10 and 15 and cage change at P5, 12 and 19. MS did not affect maternal weight [g], relative pup weight [g/n] or pup survival.. Values represent group means ± standard errors.

	control			MS		
**Dam weight [g]**						
P5	32.25	±	0.37	32.02	±	0.58
P10	34.81	±	0.50	33.96	±	0.44
P15	35.08	±	0.38	34.94	±	0.48
**Relative pup weight [g/n]**						
P5	3.22	±	0.08	3.18	±	0.09
P10	5.29	±	0.11	5.29	±	0.14
P15	7.12	±	0.16	6.85	±	0.15
**Pup survival [%]**						
P0-P15	92.82	±	2.34	94.51	±	2.01

Supplementary Table 2. Body-weight of control offspring and offspring that were exposed to maternal separation (postnatal day 24–68). Lifelong inactivation of tryptophan hydroxylase 2 (*Tph2*) decreased body-weight [g] when compared to *Tph2*$^{+/-}$ and *Tph2*$^{+/+}$ offspring, independent of maternal separation (MS) ($p<0.001$). Values represent group means ± standard errors (n=8-18). Grey tabs indicate the average weight per *Tph2* genotype, independent of condition.

	P24±2			P45±2			P68±4		
**control *Tph2*$^{+/+}$**	14.0	±	0.6	24.1	±	0.6	27.3	±	0.6
**MS *Tph2*$^{+/+}$**	15.0	±	0.5	24.3	±	0.6	27.3	±	0.6
***Tph2*$^{+/+}$**	14.4	±	0.4	24.2	±	0.4	27.3	±	0.4
**control *Tph2*$^{+/-}$**	14.7	±	0.4	24.2	±	0.5	27.3	±	0.6
**MS *Tph2*$^{+/-}$**	14.2	±	0.5	24.5	±	0.4	27.4	±	0.5

***Tph2*+/-**	14.5 ± 0.3	24.3 ± 0.3	27.3 ± 0.4
**control *Tph2*-/-**	8.6 ± 0.7	21.2 ± 0.6	24.9 ± 0.5
**MS *Tph2*-/-**	9.1 ± 0.5	20.9 ± 0.6	24.7 ± 0.5
***Tph2*-/-**	8.9 ± 0.4	21.1 ± 0.4	24.8 ± 0.3

2.2 Behavior

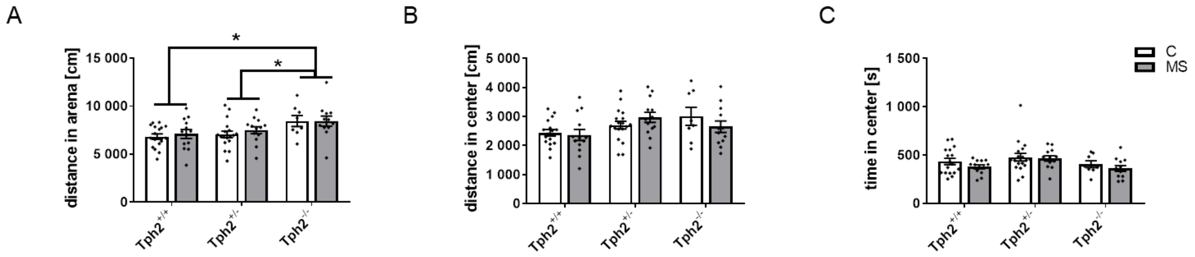

Supplementary Figure 1. Activity indicated by the total distance moved in the open-field arena. (A) total distance covered in the open-field (OF) arena was affected by the tryptophan hydroxylase 2 (Tph2) genotype, independent of maternal separation (MS). (B) distance in the center as well as (C) time in the center. Bars represent group means ± standard errors (n=8-18). * FDR corrected p<0.050. C=control

Supplementary Table 3. Overview of all statistical tests of the behavioral analysis (Table 1.XLSX).

2.3 Whole genome data

Supplementary Table 4. Overview of the statistical analysis of differentially expressed genes (Data Sheet 2.ZIP). List of differentially expressed genes (DEGs) that have been affected by gene-by-environment interaction (GE1 and GE2) of tryptophan hydroxylase 2 (*Tph2*) genotype and neonatal maternal separation (MS). Data based on sequencing counts of total RNA sequencing (group size=8). Chr=Chromosome, Bm=basemean, lg2FC=log2 fold change, lfcSE=log fold change standard error, stat=wald-statistic, lincRNA=long intergenic non-coding RNA, lncRNA=long non-coding RNA, miscRNA=miscellaneous RNA, TEC=to be experimentally confirmed, miRNA=micro RNA, snoRNA=small nuclear RNA, G=genotype, GE=gene-by-environment interaction

Supplementary Table 5. Overview of the statistical analysis of differentially methylated loci (Data Sheet 3.ZIP). List of differentially methylated loci (DMLs) that have been affected by gene-by-environment interaction (GE1 and GE2) of tryptophan hydroxylase 2 (*Tph2*) genotype and neonatal maternal separation (MS). Data based on sequencing counts of methyl-CpG-binding domain (MBD) capture-based enrichment sequencing (group size=6-8).Chr=Chromosome, Bm=basemean, lg2FC=log2 fold change, lfcSE=log fold change standard error, stat=wald-statistic, lincRNA=long intergenic non-coding RNA, lncRNA=long non-coding RNA, miscRNA=miscellaneous RNA, TEC=to be experimentally confirmed, miRNA=micro RNA, snoRNA= small nuclear RNA.

Supplementary Table 6. Overview of the statistical pathway analysis (Table 2.XLSX). List of terms comprising enriched terms of differentially expressed genes and genes with (nearby) methylated loci.

2.4 QC of MBD sequencing data

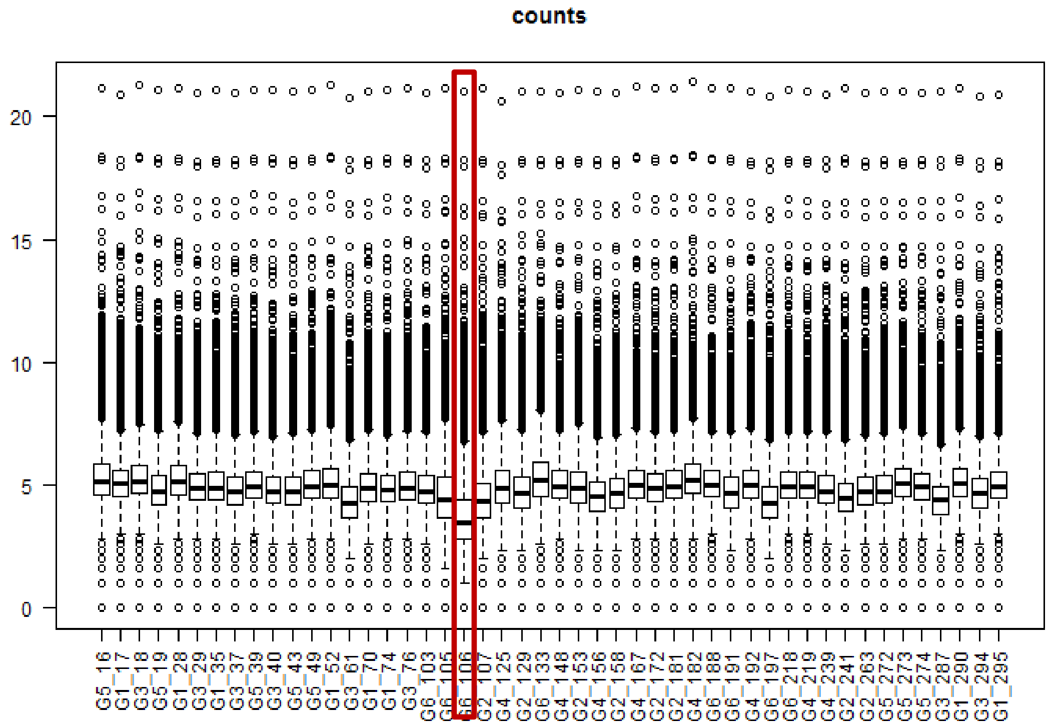

Supplementary Figure 2. Raw counts: Sample 106 showed a pronounced deviation in terms of total MBD sequencing counts

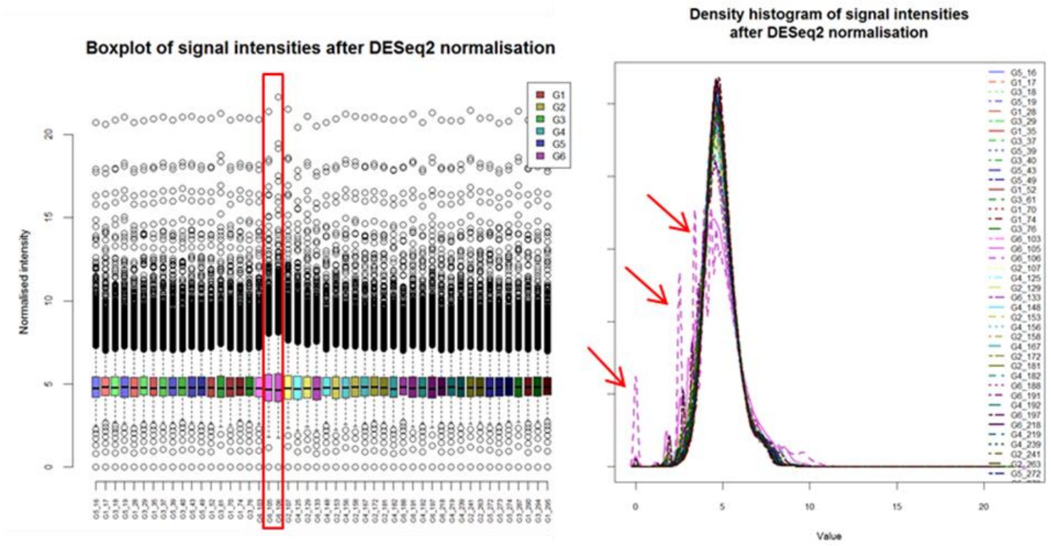

Supplementary Figure 3. Post normalization density of raw counts: sample 106 was still observably deviating, in addition, also sample 105 showed up as clearly different from other samples. Both samples are part of group 6, indicating animals with biallelic tryptophan hydroxylase 2 (*Tph2*) deficiency that were exposed to maternal separation (MS), and were excluded from analysis.

2.5 Source data

Behavior and physiological source data is comprised in **Table 3.XLSX**

Whole genome source data has been deposited in the Gene Expression Omnibus database (identifier: **GSE110330**).
